# Getting Sugar Coating Right! The Role of the Golgi Trafficking Machinery in Glycosylation

**DOI:** 10.3390/cells10123275

**Published:** 2021-11-23

**Authors:** Zinia D’Souza, Farhana Taher Sumya, Amrita Khakurel, Vladimir Lupashin

**Affiliations:** Department of Physiology and Cell Biology, University of Arkansas for Medical Sciences, 4301 West Markham Street, Little Rock, AR 72205, USA; FSumya@uams.edu (F.T.S.); AKhakurel2@uams.edu (A.K.)

**Keywords:** Golgi, glycosylation, multisubunit tethering complexes, SNAREs, congenital disorders of glycosylation

## Abstract

The Golgi is the central organelle of the secretory pathway and it houses the majority of the glycosylation machinery, which includes glycosylation enzymes and sugar transporters. Correct compartmentalization of the glycosylation machinery is achieved by retrograde vesicular trafficking as the secretory cargo moves forward by cisternal maturation. The vesicular trafficking machinery which includes vesicular coats, small GTPases, tethers and SNAREs, play a major role in coordinating the Golgi trafficking thereby achieving Golgi homeostasis. Glycosylation is a template-independent process, so its fidelity heavily relies on appropriate localization of the glycosylation machinery and Golgi homeostasis. Mutations in the glycosylation enzymes, sugar transporters, Golgi ion channels and several vesicle tethering factors cause congenital disorders of glycosylation (CDG) which encompass a group of multisystem disorders with varying severities. Here, we focus on the Golgi vesicle tethering and fusion machinery, namely, multisubunit tethering complexes and SNAREs and their role in Golgi trafficking and glycosylation. This review is a comprehensive summary of all the identified CDG causing mutations of the Golgi trafficking machinery in humans.

## 1. Introduction

Eukaryotic cells have a remarkable feature of intracellular compartments or organelles enclosed by lipid membranes. Compartmental identity is determined by a number of factors, including the membrane lipid and luminal composition as well as by the transmembrane and peripheral proteins associated with the organelle [1,2]. The secretory pathway transports cargo not only to the extracellular space for secretion but also between organelles. The endoplasmic reticulum (ER), the Golgi apparatus (GA) and the endolysosomal system are key organelles of this pathway. Proteins of the secretory pathway collectively referred to as cargo are synthesized in the ER by ribosomes. Upon completion of synthesis, the Golgi receives incoming cargo from the ER. Within the lumen of the Golgi, proteins undergo posttranslational modifications and are finally sorted into appropriate carriers targeted to their destination.

The GA is the central hub of the secretory pathway. It is composed of at least four morphologically distinct cisternae named *cis*, *medial*, *trans*, and the *trans*-Golgi network (TGN), each having its own identity. The GA functions as a station that processes and packages cargo into appropriate membrane carriers. The cargo arrives at the GA continuously and bidirectionally. Biosynthetic cargo is transported in an anterograde manner from the ER to the Golgi and then to the post Golgi compartments, while retrograde cargo is brought to the Golgi by the endosomal system. The currently accepted cisternal maturation model of Golgi transport describes that the anterograde cargo stays in the Golgi cisternae and is carried forward by the gradual maturation of the *cis*-Golgi to the *trans*-Golgi while Golgi resident proteins are cycled back to newly formed cisternae [3,4,5,6]. This way, the identity of each compartment is maintained even as the cisternal maturation goes on.

Lipids and proteins transported by the secretory pathway undergo modifications such as glycosylation within the Golgi. The different known types of glycosylation are classified according to the amino acid residue to which the carbohydrate is attached. In humans, N-glycosylation and O-glycosylation are the two main types. N-glycans are where glycans are attached to Asn in the consensus peptide sequence Asn-X-Ser/Thr, while O-glycans are where glycans are attached to hydroxyl groups of serine or threonine. N-glycosylation is a well-defined process that begins in the ER while O-glycosylation is more varied and mostly begins in the Golgi [7,8,9,10]. Protein glycosylation includes the addition of N-linked glycans, O-linked glycans, phosphorylated glycans, glycosaminoglycans and glycosylphosphatidylinositol (GPI) anchors to peptide backbones as well as C-mannosylation of tryptophan residues [11]. The Golgi houses the majority of the glycosylation machinery within its cisternae. The glycosyltransferases, glycosidases together with their accessory machinery, modify and process glycans as they traverse the Golgi. At the TGN, fully processed glycoconjugates are packaged into carrier membranes and transported to appropriate destinations.

The glycosylation of macromolecules is involved in nearly every biological process and its defects give rise to various pathologies [11,12]. Glycosylation defects affect multiple organ systems with varying severities, and they fall under the umbrella of congenital disorders of glycosylation (CDG). CDGs are autosomal recessive inherited diseases due to mutations in genes involved in glycosylation [13,14]. CDGs are further subdivided into type I (CDG-I) and type II (CDG-II) [15,16]. CDG-I is due to defects in the transfer of preassembled dolichol-glycan in the ER (or cytosol). Defects in glycan processing in the Golgi give rise to CDG-II. Formerly, a CDG diagnosis was made only based on the isoelectric focusing of serum transferrin which only detected N-glycosylation defects. Nowadays, glycan mass spectrometry and/or ApoCIII IEF are often used to complement transferrin IEF testing [17].

## 2. Glycosylation Enzyme Compartmentalization

Apart from the glycosylation enzymes, the Golgi glycosylation machinery also includes ion channels and sugar transporters that maintain intraluminal pH and bring in cofactors and charged monosaccharides required for glycosylation. The key quality control for glycosylation is the proper cisternal compartmentalization of the glycosylation machinery [9,18,19,20]. Glycan processing/addition occurs in the sequence in which the enzymes encounter their substrates. As the *cis*-Golgi matures, eventually becoming the TGN and carrying cargo forward, the correct compartmentalization of the glycosylation machinery must be maintained within the framework of cisternal maturation. At the TGN, the cargo is sorted into appropriate anterograde carriers targeted to various destinations.

All Golgi glycosylation enzymes are transmembrane (TM) proteins and the majority of them are type II TM proteins. They possess a catalytic C-terminal domain in the lumen, a luminal stem region, a transmembrane domain (TMD) and a short N-terminal cytosolic tail. Evidence suggests that the cytoplasmic tail and TMD of Golgi enzymes, cytosolic adaptor and coat proteins, and membrane lipid composition play a role in the sorting and correct cisternal localization of the glycosylation machinery. The current models for sorting and retention at the Golgi have been described by Banfield et al. in [21,22] and more recently by Welch et al. in [23]. Here we summarize the retention and recycling mechanisms that have been described.

“Kin recognition” was the earliest proposed model for enzyme retention in the Golgi [24]. Kin oligomerization was first identified in the medial Golgi enzymes N-Acetylglucosaminyltransferase-1 (GlcNAcT1/MGAT1) and Mannosidase-2 (ManII/MAN2A1) [25]. These enzymes interact with each other via their TMDs and stem regions. Because enzyme oligomers would be too big to enter transport vesicles exiting the Golgi, they are retained in the Golgi. The enzyme α-2,6-sialyltransferase-1 (ST6Gal1) is believed to possess trans Golgi localization signals in its cytoplasmic tail, TMD and stem region because mutations in these regions lead to the secretion of the enzyme [26,27]. ST6Gal1 not only forms homodimers but also heteroligomerizes with β-1,4-Galactosyltransferase-1 (B4GALT1) [28]. All the currently known oligomers of the known Golgi enzymes in various glycosylation pathways have been described by de Graffenried et al. in [29].

Transmembrane domain-dependent sorting relies on compatibility between the TMD and membrane thickness [30]. Membrane thickness is believed to gradually increase from the ER to the PM due to different lipid compositions. Phospholipids are enriched in ER and Golgi membranes, while the enrichment of sphingolipids and sterols gradually increases in the TGN and post-Golgi compartments with the highest abundance in the PM. An extensive and detailed comparison of the TM proteins found that ER and Golgi residents have shorter TMDs compared to post-Golgi residents and bulkier residues were distributed closer to the exoplasmic side of the bilayer TMDs [31]. Simply stated, shorter TMDs reside in thinner membranes. Thus, Golgi enzymes are kept from entering the post-Golgi compartments by the mere incompatibility of their short TMDs in thicker membranes.

Golgi enzymes can cycle between the Golgi cisternae [32,33] as well as between the Golgi and ER [34], and the majority of recycling occurs in COPI-coated vesicles. Yeast α-1,6-mannosyltransferase (Mnn9p) is one such example [35,36]. This *cis*-Golgi enzyme is retrieved in COPI vesicles at the TGN [35]. A few other glycosylation enzyme complexes such as the Man-Pol-I (Mann9p and Van1p) and Man-Pol-II (Mnn9p, Hoc1p, Anp1, Mnn10p, and Mnn11p), in yeast, cycle through the ER in COPI vesicles. Selective sorting into retrograde COPI-coated carriers either occurs via direct interaction between the cytosolic tails or via an adaptor. Sorting signals—KKXX or RXR on ER residents [37] or φ(K/R)XLX(K/R) on some *cis*-Golgi enzymes—directly interacting with COPI subunits have been identified on the cytoplasmic tails of a subset of enzymes [38,39,40]. Vps74 is a COPI interacting protein that was identified as a protein essential for the proper localization of a subset of glycosylation enzymes in yeast [41]. Vps74 recognizes the ([F/L][L/I/V]XX[R/K]) peptide motif on the cytoplasmic tails of a subset of glycosyltransferases [42]. GOLPH3 and GOLPH3L are human homologs of Vps74. They have an affinity for PI4P enriched in the trans-Golgi and recognize membrane-proximal polybasic residues on the cytoplasmic tails of a subset of Golgi enzymes, including ST6Gal1 and N-acetylgalactosaminyltransferase-2 (GALNT2) [33,43]. About half the number of enzymes involved in glycosphingolipid synthesis have an LXX[R/K] consensus motif recognized by GOLPH3 on their cytosolic tails [44].

These mechanisms of enzyme compartmentalization within the Golgi are not mutually exclusive.

## 3. Membrane Trafficking Machinery

**Figure 1:**
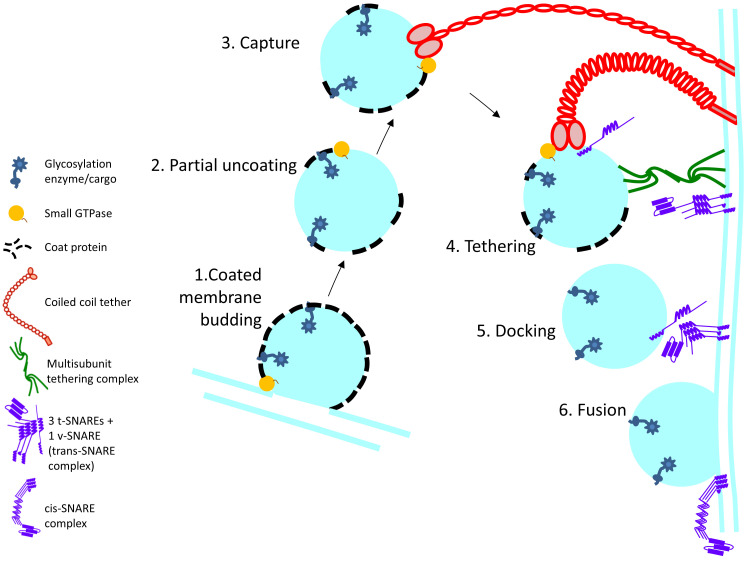
Schematic outline of the steps involved in Golgi enzyme recycling: 1. Membrane budding at the donor membrane is initiated by recruitment of coat proteins which directly or indirectly interact with cargo (glycosylation enzyme) 2. and 3. After budding, the coat protein partially falls off and the vesicle is captured by long coiled-coil tethers. 4. Among other interactions, MTCs interact with the SNAREs on the vesicle and target membrane, thereby facilitating SNARE alignment 5. and 6. Priming of the SNAREs and formation of the *trans*-SNARE complex leads to vesicle fusion at the target membrane and cargo delivery.

The membrane trafficking machinery moves the cargo in membrane-enclosed carriers to various destinations within the cell or releases secretory cargo at the plasma membrane. Every compartment in the secretory pathway is equipped with its own set of molecular players for transport. This machinery can be broadly categorized based on its function as budding, tethering and fusion machinery (Figure 1). The events of budding and fusion are controlled by a set of GTPases that modulate the activities of the trafficking machinery. Coat proteins together with cargo adaptors bind to the cargo, recruit them into specialized domains and bud these membrane domains off in the form of small vesicles or tubules [45,46]. COPI, COPII and Clathrin are the three known coat proteins. COPI is involved in the intra-Golgi and Golgi to ER retrograde trafficking, COPII is involved in the ER to Golgi anterograde trafficking and Clathrin is involved in TGN-plasma membrane bidirectional transport. Arf1 and Sar1 are small GTPases that are involved in vesicle formation. Membrane localized active Arf1-GTP recruits COPI and clathrin components while Sar1-GTP recruits the COPII components [47]. Membrane curvature is triggered by dimerization of Arf1-GTP [48]. Fission is thought to be driven by the interaction between the GTPases’ amphipathic helices and membrane lipids [48]. The membrane-enclosed cargo carrier is then tethered to its specific target membrane. Alignment of the fusion machinery on the two opposing membranes results in membrane fusion and the delivery of cargo to its destination. The function and intricate interactions between all the factors at play in cellular transport are yet to be fully understood. For this review, we elaborate on the tethering and fusion machinery at the Golgi and the disorders that arise due to genetic mutations in these trafficking factors.

### 3.1. Tethers (MTCs and Coiled-Coil Tethers)

Based on structure and function, tethers fall into two broad categories, namely coiled-coil tethers and multisubunit tethering complexes (MTCs).

#### 3.1.1. Coiled-Coil Tethers

**Figure 2:**
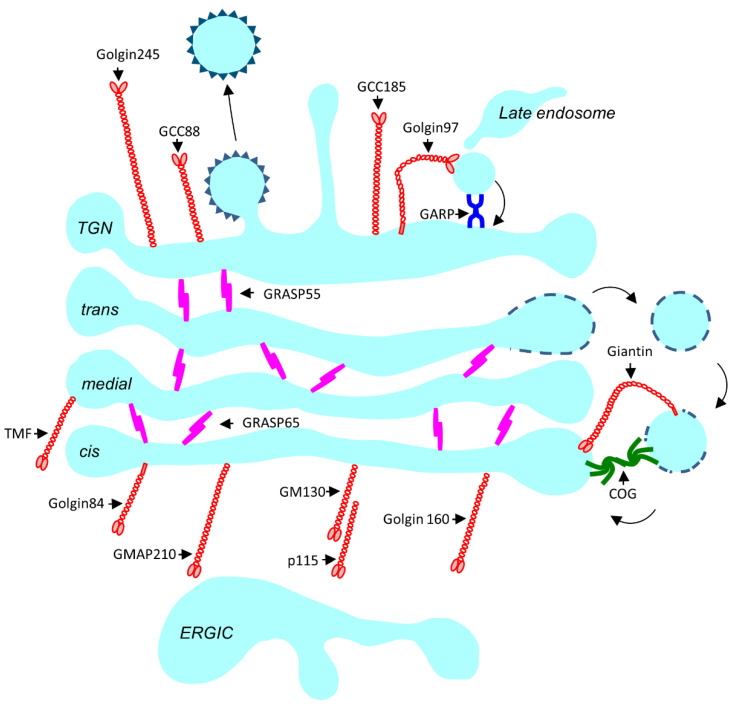
Schematic outline of localization of the coiled-coil tethers and the MTCs at the Golgi. Coiled-coil tethers are depicted in red. Golgi stacking proteins GRASP55 and GRASP65 are depicted in pink. The conserved oligomeric Golgi (COG) complex is the MTC that operates at the Golgi and is depicted in green, while the (Golgi associated retrograde protein) GARP complex operates at the TGN and is depicted in purple.

Coiled-coil tethers are structurally similar 100–200 nm long homodimers with large globular heads. Their length extends into the cytosol enabling the long-range capture of incoming cargo carriers and brings them close to their target membrane. At the Golgi, they may also function as stacking proteins holding the cisternae together (for a more in-depth review see [49] (Figure 2). The majority of the coiled-coil tethers that reside at the Golgi are called Golgins (see Table 1 for a complete list) (Figure 2). The *cis*-Golgins (USO1 (p115), GOLGA2 (GM130), TRIP11 (GMAP210), GOLGA3 (Golgin160), and GO45 (Golgin45)), and *trans*-Golgins (GOLGA1 (Golgin97), GOLGA4 (Goglin245), GCC1 (GCC88), GCC2 (GCC185), and TMF1 (TMF)), are soluble peripheral membrane proteins. GOLGB1 (Giantin), GOLGA5 (Golgin-84) and CUX1 (CASP) have a transmembrane domain at their carboxyl termini and localize at the rims of the Golgi stack. GORASP1 (GRASP65) and its homologue GORASP2 (GRASP55) are also Golgi matrix proteins but are not classified as Golgins due to their structural differences.

GOLGA2 (GM130 or Golgin95): This protein plays an important role in the development of vertebrates and invertebrates. Targeted or global depletion of GM130 in Drosophila, zebrafish and mice have shown developmental effects in the nervous and skeletal system [50,51,52]. Recently, GOLGA2 KO in mice has also been implicated in liver and lung fibrosis due to increased autophagy [53]. Two cases of GOLGA2 mutations have been identified in the human population. Both cases have neuromuscular disorders, including developmental delay, muscular dystrophy, seizures and microcephaly [51,54]. In a 2019 study, a lower level of GM130 expression was shown to cause aberrant O-glycosylation and was linked to Berger’s disease, also known as IgA neuropathy [55]. Though GOLGA2 CDG causing mutations in humans have not been reported, studies from cell lines do point to the essential role of GM130 in maintaining the Golgi structure and proper glycosylation [56,57].

TRIP11 (GMAP210): Early studies on GMAP210 point to its function in Golgi cisternal organization and positioning by anchoring microtubules at the *cis*-Golgi [58,59]. Its role in maintaining the Golgi stacks was also confirmed in HeLa cells wherein TRIP11 KD caused fragmentation and vesiculation of the Golgi [60]. It is required for proper anterograde and retrograde trafficking at the ER–Golgi interface [61]. It was also shown that GMAP210 can directly capture intra-Golgi vesicles [62]. Mutations in the thyroid hormone receptor interactor 11 (TRIP11) gene encoding the Golgi-associated microtubule-binding protein 210 (GMAP210) in the human population give rise to a lethal neonatal skeletal dysplasia termed as achondrogenesis (ACG1A) type IA or the much milder nonlethal odontochondrodysplasia (ODCD) depending on whether the mutation leads to a complete or partial loss of function [63,64,65,66,67]. In ACG1A and ODCD, Golgi disruption impairs the posttranslational processing and secretion of several extracellular matrix (ECM) components [64]. A mouse model bearing a nonsense mutation in TPIP11 with a complete loss of GMAP-210 resulted in ACG1A with fetuses showing an absence of mineralization. This affected chondrocyte differentiation, made them apoptotic, and reduced ECM production. Detailed analysis of cellular defects in the chondrocytes and osteoblasts revealed ER stress, vesicle aggregation and abnormal Golgi morphology. Studies on the glycosylation status in TRIP11 mutant mice fibroblasts using lectin staining confirmed incomplete glycan processing in the Golgi. Proteoglycan synthesis also differed in the mutant chondrocytes. The same study also investigated a 27-week-old human fetus with lethal ACG1A and found a similar phenotype as they did in their mouse model [63]. In hypomorphic mutations in TRIP11 causing ODCD, the milder counterpart of ACG1A, patient fibroblasts showed some degree of Golgi-localized GMAP210, which was completely absent in the ACG1A patient’s and the Golgi morphology was mostly normal. Patient fibroblasts also secreted reduced levels of ECM proteins. Both ACG1A and ODCD causing TRIP11 mutations have similar negative effects on glycosylation and proteoglycan modification of the ECM component decorin [64].

GOLGA3 (Golgin 160): Like GMAP210, Golgin160 is also localized to the *cis*-Golgi and functions in Golgi positioning and structure. It recruits the microtubule motor dynein to the Golgi [59,68]. Similar to the GMAP210 KD effects in HeLa cells, siRNA-induced KD of Golgin160 in the same cell line resulted in Golgi fragmentation and the dispersal of Golgi mini stacks [59]. A missense mutation in GOLGA3 has been linked to autism [69].

USO1 (p115): p115 belongs to the golgin family of tethers. It is involved in transport between ER and Golgi. At the *cis*-Golgi it interacts with the golgins, GM130 and Giantin as well as the SNAREs, BET1 and Sec22b [70,71,72]. The golgin, p115, was discovered as a protein that stimulated the proper glycosylation of the cargo protein VSVG in the in vitro intra-Golgi trafficking setup [73]. It was later shown to play an important role in the formation of pre-Golgi ERGIC intermediates [74]. Early studies suggested that p115 is recruited by Rab1 to COPII vesicles budding from the ER where it interacts with COPII vesicle-associated SNAREs [75]. It interacts with the STX5 SNARE partners GOSR1/BET1L/YKT6 and GOSR2/BET1/Sec22b [76] and p115 is essential for cisternal stacking and cisternal regrowth, facilitating the initial tethering of Golgi cisternae [77]. Moreover, it has a vital role in the biogenesis and maintenance of the Golgi as it interacts with the COG complex and participates in vesicular trafficking at the Golgi [78]. Although the depletion of p115 in tissue culture cells leads to glycosylation defects [79], CDG-causing mutations have not been reported so far.

GOLGB1 (Giantin): Giantin is the largest and well-characterized *cis*-golgin. It is anchored at the *cis*-Golgi by its transmembrane domain and either has a short or no luminal domain at its C-terminus [80,81]. Regulation of the Golgi architecture is one of its key functions. Apart from p115, Giantin interacts with Rab1, Rab6, and GM130. Loss of Giantin can accelerate the anterograde transport instead of blocking it [76,81,82,83,84]. While Giantin may be dispensable in maintaining Golgi structure, its depletion inhibits lateral tethering between the cisternae [83,84]. GOLGB1 loss-of-function zebrafish [82] and mouse [85] models revealed that Giantin has specific functions in protein glycosylation and tissue morphogenesis. The expression of Golgi-resident glycosyltransferases like GALNT3 is altered due to a loss of Giantin [82].

GOLGA5 (Golgin-84): Human golgin-84 is well conserved in evolution with orthologs in plants, but not fungi. Golgin-84 directly interacts with another Golgi tether, CASP, [86] and the COG7 subunit of the COG complex [87], and its siRNA-driven depletion in HeLa cells causes glycosylation defects [88]. Surprisingly, GOLGA5 is dispensable for mouse embryonic development and postnatal survival [89], indicating the redundancy in Golgi coiled-coil tethers. It was also demonstrated that Golgin-84 can directly capture intra-Golgi vesicles carrying glycosylation enzymes via its N-terminus [62].

GOLGA1 (Golgin-97): This protein was identified as an autoantigen in patients with Sjögren’s syndrome [90]. Golgin-97 is localized at the TGN where it tethers endosome-derived vesicles [91]. It associates with the centrosome [92], plays a role in the retrograde trafficking from endosomes to TGN [93] and its silencing leads to Golgi fragmentation [94]. This protein has garnered particular interest in the field of virology due to its requirement in poxvirus replication [95].

GOLGA4 (Golgin-245): Like Golgin-97, Golgin-245 too was identified as an autoantigen in patients with Sjögren’s syndrome. It is involved in the retrograde trafficking from endosomes to TGN and contributes to Golgi positioning around the centrosome. In a HeLa cell line, siRNA-mediated depletion of Golgin-245 resulted in TGN dispersal and fragmentation of the Golgi ribbon into mini stacks [96]. Displacement of Golgin-245 from the TGN or siRNA silencing disrupts the anterograde transport of cell surface destined cargo from the TGN [97,98,99]. In a more recent study, this protein has also been shown to function in phagophore formation during autophagy [100]. It was also demonstrated that Golgin-245 could directly capture endosomal-derived vesicles via its N-terminus [62].

GCC1 (GCC88): This GRIP-domain containing TGN localized Golgin is involved in endosome to Golgi retrograde transport [101] and maintains the tubular organization of the TGN [102,103]. GCC88 KD in HeLa cells led to mislocalization of the Qc SNARE syntaxin 6 which prevented the recycling of TGN38 and CI-M6PR from endosomes to the TGN [101]. Due to impaired CI-M6PR recycling, lysosomal activity was perturbed and the lysosomal enzyme Cathepsin D’s processing was impaired [103].

GCC2 (GCC185): This is another GRIP domain-containing protein targeted to the TGN and has the dual functions of tethering vesicle and cytoskeletal organization by providing a microtubule attachment site [104]. It binds the TGN Qa SNARE STX16 [105]. Rab9 localizes GCC185 at the endosomes while Rab6 and Arl1 are suggested to localize it at the TGN [106,107,108], but Rab6 KD does not mislocalize GCC185 from the Golgi [109]. It functions in the CI-M6PRs recycling from endosomes to TGN. KD in HeLa cells fragments the Golgi [106,110]. Despite a fragmented Golgi in GCC185 KD cells, anterograde trafficking is unperturbed, but a subset of the retrograde cargo, Shiga toxin, for example, is blocked in endosomes [110].

TMF/ARA160: Tata element modulatory factor (TMF/ARA160) is a multifunctional Golgi tether that also functions as a transcriptional factor. Smg1, the yeast homologue, is recruited to the TGN by Ypt6-GTP and its deletion in yeast does not produce any trafficking defect [111,112]. TMF KD causes a dispersal of the Golgi cisternae [112], blocks endosome to TGN retrograde trafficking and mislocalizes GalNAc-T2 [113]. It directs the proteasomal degradation of Stat3, a cell growth regulator and p65 [114,115]. Interestingly TMF null male and female mice develop normally, but males are sterile with defects in the testis and epididymis. These mice produce acrosome-deficient-morphologically-defective immotile sperms, and have low testosterone levels [116,117]. It interacts with the microtubules and plays a critical role in Golgi orientation during spermatogenesis [118]. More recently, it was also found to be responsible for the transport of vesicles containing GLUT4, a receptor that drives glucose uptake in an insulin-dependent manner [119].

GRASP65 and GRASP55: The mammalian Golgi reassembly stacking proteins of 65 kDa (GRASP65/GORASP1) and 55 kDa (GRASP55/GORASP2) are two homologous Golgi transport proteins localized to the *cis*- and *medial-trans* cisternae, respectively [120,121]. GRASPs are required for the in vitro stacking of Golgi cisternae as well as Golgi ribbon formation by linking individual stacks [122,123,124]. Interestingly, a recent study reported that auxin-mediated acute depletion of GRASP55 and/or GRASP65 does not affect Golgi stack formation but causes the loss of lateral connectivity [125]. GRASP65 interacts with GM130 and GRASP55 forms a complex with Golgin-45 to regulate Golgi morphology and tethering [122,126]. Recently, a CRISPR/Cas9 mediated GRASP double knockout was reported to disperse the Golgi stack into single cisternae and tubulovesicular structures, increase protein trafficking and impair proteins and lipid glycosylation in HeLa and HEK293T cell lines [121,127]. Both the single and double GRASP knockout cell lines had reduced the cell surface binding of wheat germ agglutinin (WGA) that binds sialic acid and N-acetylglucosamine, and that of Maackia amurensis lectin (MAA), which binds α(2,3) sialic acid. In addition, LAMP1 and LAMP2 glycoproteins’ increased mobility indicated that they were not fully glycosylated [121]. Glycan mass spectrometry analysis confirmed that GRASP55 and GRASP65 depletion affects N-linked glycans in HeLa cells [127]. Furthermore, the altered cell surface binding of Shiga and Cholera toxins suggests that a GRASP knockout impairs the production and/or trafficking of glycolipids as well [121]. Besides protein and lipid glycosylation defects, GRASP depletion also causes sorting defects resulting in the production of immature Cathepsin D [127].

#### 3.1.2. Multisubunit Tethering Complexes

##### Conserved Oligomeric Golgi Complex (COG)

The conserved oligomeric Golgi (COG) complex is the major CATCHR (complexes associated with tethering containing helical rods) vesicle tethering, hetero-octameric complex in the Golgi vicinity, with subunits COG1-COG8 [128,129,130,131]. It is divided into two sub-complexes named Lobe A (COGs 1–4) and Lobe B (COGs 5–8) with an interaction between COG1 and COG8 [128,132,133,134]. The COG complex appears to interact with all types of trafficking facilitators throughout the Golgi, such as SNAREs, SNARE-interacting proteins, Rabs, coiled-coil tethers, and vesicular coats [134]. The array of protein interactions suggests that COG, generally, orchestrates sequential processes triggered by activated Rab proteins beginning with vesicle attachment and concluding with the assembly of SNARE complexes [135]. Different COG subunits assigned as different “interaction hubs” such as COG2 and COG3 act as “tether hubs,” COG2, COG3, and COG4 act as “coat and motor hubs,” COG4, COG5, and COG6 act as “Rab hubs,” and COG4, COG6, COG7, and COG8 act as “SNARE hubs.” The COG complex has two potential conformations which were uncovered by electron microscopy (EM) in fixed and unfixed conditions. When unfixed, the COG complex has an extended and seemingly flexible structure (approximately 50–75 nm long) with multiple elongated, curved arms with globular or ‘hook like’ ends, whereas when fixed, the COG complex has a more globular appearance (∼37 nm in length) with rod-like connections between the two main lobes [128,129,130,135,136]. The exact conformation of the membrane-bound COG complex is unknown; however, live-cell super-resolution microscopy studies indicate that COG lobe A is preferentially Golgi bound, while lobe B is localized on vesicles [137].

COG mainly acts as a vesicular tether in retrograde intra-Golgi trafficking and recycles Golgi resident proteins (such as glycosylation enzymes) to maintain proper glycosylation of secretory proteins [138,139,140,141]. Recent studies on a complete set of COG subunit KOs in HEK293T cells, using a CRISPR-Cas9 approach, provided further insights into the COG’s function at the Golgi [142]. All COG deficient cells had glycosylation defects implicating the role of both lobes of the COG complex in maintaining glycosylation fidelity. Furthermore, each COG subunit-deficient cell line had defects in Golgi morphology, retrograde trafficking, sorting, and secretion, and accumulated enlarged endolysosomal structures (EELSs) [143,144]; however, the severity of these defects varied among subunits [142].

Almost one-third of the patients with congenital defects in the Golgi glycosylation have mutations in one of the subunits of the COG complex and this type of CDG is termed COG-CDG [10,145] (Table 1). Over a hundred individuals with 31 different mutations in different COG subunits (except COG3) have been identified to date, presenting heterogeneous clinical features and cellular phenotypes. COG-CDGs are rare and incurable diseases that belong to a group of autosomal recessive disorders and multi-systemic disorders with several distinguishable symptoms that include global developmental defects, including dysmorphic features, microcephaly, and failure to thrive. These deficits are often accompanied by liver and neurological impairment [13]. Lobe A (COG) mutations are proposed to be more deleterious than lobe B mutations [146], but it is still unclear because of the small number of known mutations and the lack of robust data on these patients. It has been reported that COG1 and COG4 patients present milder problems in comparison to COG7 and COG8 patients [147]. Among all the COG-CDGs, COG7 mutation is the most severe and COG6-CDG is nearly severe as 50% of the patients died within the first two years of life [148]. Neurological symptoms are most common in COG5 and COG8-CDG patients, while growth retardation is predominant in COG1 and COG7-CDG patients [145,149]; however, known COG mutations do not cause embryonic lethality suggesting that there is some degree of tolerance to COG malfunctions during embryonic development [131]. Clinically, prominent incomplete galactosylation and sialylation has been found in patients of COG-CDGs for variants of both the lobe A and B subunits [14]. Recent comprehensive reviews from ours and other groups have summarized the cellular and clinical manifestations of all the reported COG mutations up to 2020 [13,14,131].

##### Golgi-Associated Retrograde Protein Complex (GARP)

The GARP complex is another Golgi-localized multisubunit vesicle tethering complex of the CATCHR family [150,151,152]. Its subunits, VPS52, VPS53, and VPS54, were first identified in a Saccharomyces cerevisiae genetic screen which was designed to find new vacuolar protein sorting (VPS) genes essential in Carboxypeptidase Y (CPY) sorting [153]. These novel trimeric protein complexes were initially named as VPS fifty-three (VFT) [111]. Later, a fourth subunit, VPS51, was discovered in another genetic screen, a mutation of which has a similar defect in CPY sorting [154,155]. A co-immunoprecipitation experiment demonstrated that VPS51 belongs to the same complex as VFT [154]. Hence, this complex is composed of four different subunits named VPS51, VPS52, VPS53, and VPS54, and each of these four subunits forms an obligatory 1:1:1:1 complex [156,157]. All of these GARP subunits require the coiled-coil motif for assembly into the complex [154,155,158].

The GARP complex is evolutionary conserved, with homologues discovered in worms, flies, mammals, and plants [150,159,160,161]. GARP is localized at the TGN. In human cells, the small GTPases ARL5 and ARFRP1 localized at the TGN are responsible for the recruitment of GARP [162]. The GARP complex shares VPS51, VPS52, and VPS53 with another tethering complex-endosome associated recycling protein (EARP) whereas VPS50 is a unique subunit of EARP and VPS54 is a unique subunit of GARP [163]. The VPS54 subunit of the GARP complex has its N-terminal domain crucial in assembly and stability of the GARP complex, whereas the C-terminal domain mediates the localization to an early endocytic compartment [164].

GARP functions in the recycling receptors for lysosomal hydrolase precursors, transmembrane proteins, and SNAREs to the Golgi [157,165,166]. Depletion of the GARP subunit blocked the retrograde trafficking of the lysosomal hydrolase receptor Mannose-6-phosphate receptor, TGN protein TGN46, and the B subunit of the Shiga toxin. As a consequence of the blockade in retrograde trafficking, there is missorting of the precursor of the acid hydrolase Cathepsin D [166]. GARP is also essential for the maintenance of cholesterol and sphingolipid homeostasis, disruption of which can lead to neurodegenerative diseases known as “progressive cerebello–cerebral atrophy type 2” and “Hereditary spastic paraparesis” [167,168,169,170]. Auxin-induced acute depletion of yeast GARP subunits resulted in the missorting of plasma membrane proteins of two different functional groups, amino-phospholipid flippases and cell wall biosynthesis proteins [171].

Exome sequencing of a 6-year-old Caucasian female identified compound heterozygous mutations (c.1468C > T and c.2232delC) in the gene encoding the VPS51 resulting in a neurodevelopmental disorder [172]. The VPS52 subunit of GARP has shown to interact with Parkinson’s disease kinase LRRK2 (Leucine-rich repeat kinase 2) and contributes to intracellular trafficking by stabilizing the GARP–SNAREs complex formation [173]. Depletion of GARP exacerbates the neurodegeneration in C. elegans [173] (Table 1).

In mice, a null mutation in the VPS54 subunit of the GARP complex causes embryonic lethality [174,175]. The “wobbler mouse” phenotype is caused by a spontaneous missense mutation L967Q near the C terminus of VPS54. The “wobbler mutations” characteristics, such as cellular transport abnormalities, progressive motor neuron degeneration, and neuroinflammation, are quite similar to human Amyotrophic lateral sclerosis, although there are no known GARP genetic abnormalities associated with human ALS patients. The “wobbler mutation” causes the VPS54 protein, as well as the whole GARP complex, to become unstable and impairs retrograde vesicular trafficking. This affects spermiogenesis and also leads to spinal muscular atrophy [176,177,178,179,180]

GARP is structurally and functionally similar to other multisubunit tethering complexes such as COG, Dsl1, and Exocyst [181]. Although the COG dysfunctions are associated with CDG, the connection between GARP function and Golgi glycosylation was discovered only recently. A case study of a 6-year-old patient carrying a mutation in VPS51 showed abnormal glycosylation testing, and her phenotype had substantial similarity with that seen in CDGs. Additionally, a significant reduction in the level of N- and O-glycosylated protein TGN46 were found in the patient’s fibroblasts. Moreover, immunostaining of LAMP2, a lysosomal protein in VPS51 mutated patient fibroblasts, demonstrated enlarged lysosomes, and immunostaining of the Mannose-6 phosphate receptor showed dispersed cytoplasmic staining in the patient fibroblasts, which is in contrast to bright juxtanuclear staining in control fibroblasts [172]. Consistently, deletion of the GARP complex subunits in tissue culture cells revealed defects in N- and O-glycosylation. These defects in the GARP complex alter the total level of the *cis*-Golgi protein GPP130, *medial-trans*-Golgi TMEM165, and the TGN-resident TGN46 Golgi proteins. In addition, lysosomal glycoprotein LAMP2, showed hypermobility on the SDS-PAGE of GARP deficient cells, indicating protein misglycosylation [143,182]. Misglycosylation of these Golgi glycoproteins can occur as a consequence of the reduction in Golgi enzymes that are responsible for N- and O- glycosylation [9]. Indeed, the protein level of the N-glycosylation Golgi enzymes α-1,3-mannosyl-glycoprotein 2-β-N-acetylglucosaminyltransferase (MGAT1), B4GALT1, ST6GAL1 and the O-glycosylation enzyme GALNT2 was reduced in GARP subunit depleted cells. Furthermore, a retention using selective hooks (RUSH) assay showed that B4GALT1 is not retained at the Golgi complex in GARP-KO cells but missorted to the endolysosomal system. The Golgi-resident enzyme ST6GAL1 was also not found to be retained in the Golgi in GARP impaired cells. This study also demonstrated that this defect in Golgi enzymes only occurs in GARP-deficient cells and not in EARP-deficient cells. The malfunction, mislocalization, and reduced stabilization of Golgi enzymes associated with a glycosylation defect were found to be rescued upon expression of the missing GARP subunit. Taken together, GARP plays a crucial role in normal Golgi glycosylation by mediating the maintenance of the Golgi glycosylation machinery [182]. While neurodevelopmental disorders are common in GARP mutated patients, it is crucial to investigate and characterize the role of the GARP complex in different cell types. The mechanisms of GARP function need to be further uncovered to elucidate how GARP is involved in the misglycosylation of proteins.

#### 3.1.3. SNAREs

**Figure 3:**
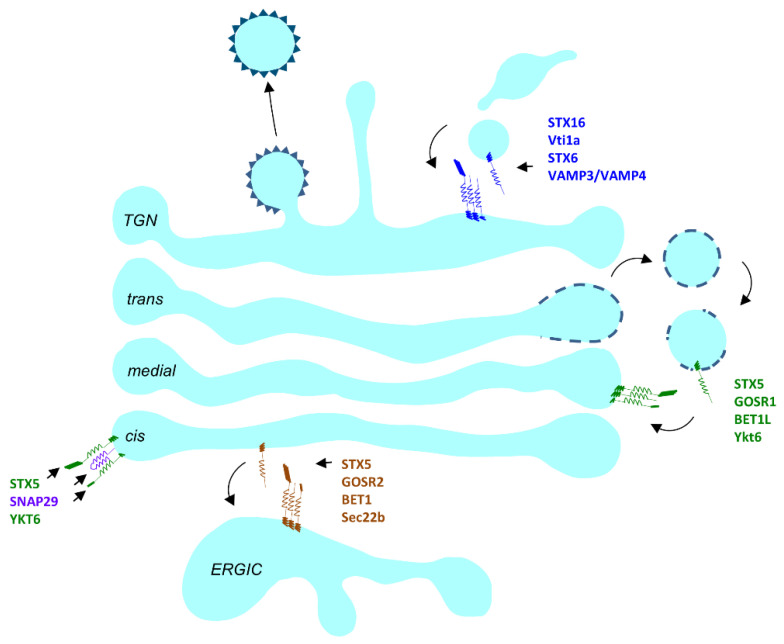
Schematic representation of the SNARE complexes participating at the Golgi. The STX5/GOSR1/BET1L/YKT6 (green) Golgi SNARE complex operates throughout the Golgi. STX5/SNAP29/YKT6 is another SNARE complex. STX5/GOSR2/BET1/Sec22b operates between the ER/ERGIC and Golgi. The STX16/Vti1a/STX6/VAMP3 or VAMP4 (purple) operates at the TGN.

SNAREs (SNAP Receptors) complete the final step of docking and fusion of the carrier membrane at its target (Figure 3). There are a total of 36 known SNAREs in humans that are present throughout the secretory pathway and confer a degree of compartment identity. Upon vesicle docking at its target membrane, usually, a total of three or four SNAREs (bearing 4 SNARE motifs) on opposing membranes align, intertwine and form a hetero-oligomeric, parallel α helical complex. The SNARE motif–a distinctive feature of all SNAREs–is a coiled structure composed of about 60–70 amino acid residues. At the center of the SNARE motif lays a glutamate or arginine residue, leading to the categorization of SNAREs as Q and R SNAREs [183]. Q SNAREs are further subcategorized into Qa, Qb and Qc based on protein profiling [184]. Typically, each Qa-, Qb-, Qc- and R-SNARE provides one SNARE motif to the SNARE complex. As the name suggests, Qbc SNAREs possess a Qb and Qc SNARE motif. These SNAREs contribute two SNARE motifs to the four-helix SNARE complex. SNARE motifs align from the N to C terminus and form an extremely stable parallel α helical complex [185]. Aligned SNAREs on opposing membranes are termed *trans*-SNARE complexes. The energy released upon the formation of the *trans*-SNARE complex drives membrane fusion. After the initiation of membrane fusion, the SNARE complex then resides on the same membrane and is termed a *cis*-SNARE complex. This transition of the SNARE complex from *trans* to *cis* is thought to play an important role in the creation and enlargement of the fusion pore [186].

SNARE complexes have to be disassembled to participate in the next round of fusion. This is an enzymatic and energy-intensive process that uses the energy of ATP hydrolysis. The enzyme NEM sensitive factor (NSF), named after it, was found to be inactivated by N-ethyl maleimide (NEM), is an AAA + ATPase. NSF is recruited to SNARE complexes by the Soluble NSF Attachment Protein (SNAP). SNAP has three isoforms- α-, β- and γ- SNAP which are ubiquitously expressed in all tissues except β-SNAP which is brain-specific. SNAP is believed to bind to the trans-SNARE complex. From there on, the NSF binds SNAP. The free energy released upon SNARE dissociation is thought to contribute to driving membrane fusion.

There are three major SNARE complexes operating in the Golgi (Figure 3). The STX5/GOSR2/Bet1/Sec22b complex mostly operates at the ERGIC compartment in mammalian cells [187]. The STX5/GOSR1/Bet1L/Ykt6 complex operates in retrograde trafficking at the Golgi [188] and the STX16/STX6/Vti1a/Vamp4 complex operates between the endosomal compartment and the *trans* side of the Golgi [189]. An additional Golgi-localized SNARE complex could be formed by STX5, SNAP29 and YKT6 [190,191].

In the STX5/GOSR2/ Bet1/Sec22b complex, GOSR2 mutations have been identified in the human population. GOSR2 (GS27/membrin) allelic variants in the human population have been associated with cardiovascular diseases and myocardial infarction [192,193,194]. A loss of function mutation (c.430G > T, p.Gly144Trp) in GOSR2 was reported to cause progressive myoclonus epilepsy with early ataxia [195], and heterozygous c.430G > T and c.82C > T is associated with muscular dystrophy [196]. The effects of GOSR2 mutations on the muscular system point to its essential role in development (Table 1).

Recently a CDG causing mutation, c.163 A > G, was identified in the Qa SNARE STX5 [197]. STX5 has two isoforms, long (STX5L) and short (STX5S), encoded by different start codons. The long isoform has an ER-retrieval sequence, while STX5S is Golgi localized. The c. 163 A > G (p.M55V) is a missense mutation at the start codon for the STX5S, leading to complete loss of STX5S protein. This mutation led to spontaneous abortion or death shortly after birth due to liver failure. Clinical presentations of this CDG include skeletal deformities, dysmorphia hypotonia, hepatomegaly, and elevated cholesterol. IEF of serum transferrin and ApoCIII together indicated N- and O- glycosylation defects which were confirmed by lectin binding analysis on patients’ fibroblasts.

Despite the essential role of SNAREs in Golgi trafficking, no other CDG has been reported so far. Embryonic lethality could result in mutations going undetected; however, due to redundancy and some degree of promiscuity among SNAREs, studies using KD or KO approaches in cell lines and other model organisms have yielded only mild phenotypes. Disruption of the Qb and Qc STX5 partners GOSR1 (GS28) and BET1L (GS15), only produce mild phenotypes. Overexpression of a BET1L lacking transmembrane domain caused Golgi disruption and mislocalization of the glycosylation enzyme MANN2 [188]. YKT6, the R SNARE in the Golgi STX5 SNARE complex, lacks a transmembrane domain and is integrated into the Golgi membrane via a lipid anchor, and its deletion is lethal in yeast [198]. Ykt6 has an essential role in Golgi organization which is disrupted when Ykt6 cannot localize to the Golgi [199].

SNAP29 is a Qbc SNARE that is reported to be membrane localized and has a cytosolic pool as well [200]. The Golgi is one of the membranes that SNAP29 is present on [190]. Among various other SNARE partners [201], it also partners promiscuously with the Golgi SNAREs STX5, STX6 [190]) and YKT6 [191], as well as the COG complex subunit, COG6 [202]. It has roles in synaptic transmission, endocytosis, cell cycle regulation and autophagy [203]. In humans, mutations in SNAP29 give rise to CEDNIK (cerebral dysgenesis, neuropathy, ichthyosis, and keratoderma) syndrome or the neuromuscular disorder PMLD (Pelizaeus–Merzbacher-like disorder) [204,205]. Zebrafish carrying CEDNIK syndrome-causing mutations have multi-systemic developmental defects [206] and the mutant male mice carrying SNAP29 mutations, in addition to phenocopying the human mutant phenotypes, are also sterile [207]. SNAP29 deletion in Drosophila disrupts the Golgi organization [208] and controlled RNAi mediated silencing leads to lethality at high temperatures [209], but in *C. elegans*, it is the endosomal system that is disrupted and not the Golgi, upon SNAP29 KD [210]. Glycosylation defects in SNAP29 mutants have not yet been investigated.

At the TGN, KD of any of the STX16 SNARE complex subunits alters endosome to TGN transport. In humans, a 4.4-kb microdeletion in the STX16 locus leads to autosomal dominant pseudohypoparathyroidism Type Ib [211]. KD of any one of the four STX16 SNARE partners at the trans-Golgi leads to Golgi fragmentation [212] yet STX16 mice are viable [213]). Both Vti1a KO and Vti1b KO mice are viable, with the latter presenting with progressive neurodegeneration [214].

## 4. Conclusions

One of the most important tasks of the GA is to carry out proper processing of its cargos. Cargos traversing the Golgi are involved in diverse physiological roles. These cargos function as PM receptors, antibodies, ligands, signaling molecules including neurotransmitters and hormones, or as matrix proteins that function in organism structure. Hence, it is not surprising that defects in intra-Golgi trafficking result in multisystem disorders. Glycosylation disorders arising from improper glycan processing within the Golgi are categorized as CDG-II. Essentially, loss of function deletions of any component of the Golgi glycosylation machinery has the potential to produce a CDG, yet CDG-II is a rare disease. The clinical presentations and cellular phenotypes of CDGs are heterogeneous in nature which makes it difficult to compare the contributions of different tethers as well as different SNAREs.

Why do defects in Golgi tethering factors and SNAREs have an effect on glycosylation? The most likely explanation is that many of these factors are involved in capturing/tethering and fusion of intra-Golgi vesicles that recycle the Golgi glycosylation machinery. Golgi enzymes can be packaged in COPI vesicles in vitro [215], but how many types and varieties of intra-Golgi vesicles exist in different cells in vivo is unknown. Mutations in the tethering/fusion components may reduce or alter specific vesicle recognition, which will lead to the loss of recycling vesicles and a corresponding reduction in one or several components of Golgi glycosylation machinery. Another possibility is that the disruption of the Golgi structure can lead to the disruption of Golgi ion and lipid homeostasis, altering enzyme activity and retention. While coiled-coil tethers are mostly involved in maintaining Golgi structure, the Golgi MTCs, COG and GARP have been found to be primarily involved in delivering proper glycosylation enzymes by maintaining the trafficking in Golgi. The COG-CDGs have been well documented [131], and recently, our lab has demonstrated GARP’s requirement in the recycling of glycosylation enzymes [182]; however, we are yet to understand why COG and GARP mutations affect some organ systems more severely than others. A fundamental question that we are posed with is why do mutations affecting the Golgi trafficking machinery have various manifestations at the cellular, tissue, and organismal level? One would imagine that any dysfunctions of the Golgi trafficking machinery would affect Golgi homeostasis and thus glycosylation because of how intimately they are tied to each other, yet this is not the case. Certain Golgi proteins are more dispensable than others and the severity of their loss varies. Redundancy among the Golgi proteins could be one possible explanation.

An in-depth understanding of the contribution of the Golgi trafficking machinery will greatly benefit the development of therapeutic approaches for patients with life-threatening mutations. In the past, patient fibroblasts have served as a good cellular model for studying the disease phenotype, but this approach has many limitations. The main caveat with fibroblasts-based studies is a potential heterogeneity resulting from a diverse genetic background of the patients. In addition, fibroblast cell physiology may not reveal the specific defects manifested in nervous, ocular, bone, and other tissues severely affected in COG patients. To study COG mutations more elaborately, cell-based, vertebrate (zebrafish) and *C. elegans* models have been developed for COG4 mutations [216]. Another cell-based model for COG4-CDG disease has been developed recently using human RPE1 and HEK293T cell lines [217]. This strategy utilizes cells with the same genetic background to directly compare the effects of different human COG4 mutations on Golgi glycosylation and trafficking. Future studies could be directed to create the cell-based model for other COG mutations revealing the cause of specific CDG. This may help to generate more data regarding the trafficking and glycosylation abnormalities in human mutated cell lines, which will lead to the development of new treatment protocols for COG-CDGs.

In this review, we have summarized human diseases as well as the effects of experimental manipulations with the Golgi trafficking machinery and tried to shed light on the contribution of vesicular tethers and SNAREs in Golgi glycosylation (Table 1). While recent studies have made significant progress in delineating the trafficking machinery, it is difficult to outline the exact mechanism by which Golgi trafficking factors control glycosylation in specific cells and tissues. Newer approaches using inducible silencing of target proteins will certainly provide a better resolution into the intricate spatial and temporal coordination among the trafficking players to achieve proper glycosylation. Furthermore, similar approaches and other precise gene editing manipulations in the early embryos of various model organisms will provide invaluable insights into the role of the Golgi trafficking machinery in development.

**Table 1:** Golgi tethering factors and SNAREs.

Gene	Alternative Names	Biological Role	Glycosylation Defects If Depleted	Known Human Mutations	References
GOLGA1	golgin-97	Endosome to TGN retrograde transport	No	-	[90,94]
GOLGA2	GM130	Maintenance of Golgi structure and ER to Golgi traffic	Yes (‘O’ glycosylation defects)	c.2251 C > T (p.Gln751Ter) c.629G > A (p.Arg210His) c.1266_1269del (p.Glu423Argfs*6)	[52,55,218]
GOLGA3	golgin-160	Maintain Golgi integrity and trafficking of plasma membrane protein	No	-	[219,220]
GOLGA4	golgin-245	Regulatory transport from TGN to plasma membrane	No	-	[100,221]
GOLGA5	Golgin-84	Tethering of intra-Golgi vesicles	Yes	-	[87,222]
GOLGA7	GCP16	-	No	-	[223]
GOLGB1	Giantin, GCP364	Maintaining Golgi structure and intra-Golgi retrograde trafficking	Yes (both ‘N’ and ‘O’ glycosylation defects)	(Golgb1ivs9+1G > A)	[81,85,224,225]
GCC1	GCC88	Efficient retrograde transport of cargo from the early endosomes to the TGN			[62,102]
GCC2	GCC185	Endosome-to-Golgi transport and maintenance of Golgi structure			[110,226]
TRIP11	GMAP210	Cisternal organization and anterograde, retrograde trafficking at ER-Golgi interface	Yes (‘N’ glycosylation defects)	c.5003T→A (p.L1668X)	[63]
TMF1	ARA160	Golgi organization			[118]
CUX1	CASP	Forms a complex with Golgin 84 and tethers intra-Golgi recycling vesicles			[86,227]
USO1	p115	ER to Golgi trafficking	Yes		[228]
GO45	Golgin-45, BLZF1	Golgi structure maintenance and secretion	-		[126]
GORASP1	GRASP65	Maintaining the Golgi structure and protein trafficking, glycosylation	Yes		[229,230]
GORASP2	GRASP55	Maintaining the Golgi structure and protein trafficking, glycosylation	Yes		[123]
COG1	KIAA1381, LDLB	Intra-Golgi retrograde trafficking	Yes (‘N’ Glycosylation defect)	2659–2660insC (p.P888fsX900) c.1070 + 5G > A	[145,231,232]
COG2	LDLC	Intra-Golgi retrograde trafficking	Yes (‘N’ and ‘O’-glycosylation defect)	c.701dup (p.Y234*), c.1900 T > G (p.W634G)	[231,233]
COG3	hSec34	Intra-Golgi retrograde trafficking	Yes		[234]
COG4		Intra-Golgi retrograde trafficking	Yes (‘N’ and ‘O’-glycosylation defect)	c.2185C > T (p.R729W) 16q22 deletion, c.697G > T (p.E233X) [de novo], c.2318 T > G (p.L773R), c.1546G > A or c.1546G > C (p.G516R)	[128,147,216,235]
COG5	GOLTC1, GTC90	Intra-Golgi retrograde trafficking	Yes (‘N’-glycosylation defect)	c.1669-15 T > C, c.556_560delAGTAAinsCT (p.S186_K187delinsL), c.1856 T > C (p.I619T) c.95T > G (p.M32R), c.2518G > T (p.E840X), c.189delG (p.C64Vfs*6), c.2338_2340dupATT (p.I780dup), c.1780G > T (p.V594F), c.1209delG (p.M403IfsX3), c.2324C > T (p.P775L), c.330delT (p.V111Lfs*22), c.1290C > A (p.Y430X), c.2077A > C (p.T693P), c.2324C > T (p.P775L), c.1508dup (p.G505Wfs*3)	[236,237,238,239,240,241]
COG6	KIAA1134	Intra-Golgi retrograde trafficking	Yes (‘N’-glycosylation defect)	c. 1646G > T (p.G549V), c.1167-24A > G (p.G390FfsX6), c.511C > T (p.R171*), c.1746 + 2 T > G, c.1238_1239insA (p.F414Lfs*4), c.1646G > T (p.G549V), c.785A > G (p.Y262C), c.511C > T (p.R171*), c.1746 + 2 T > G	[148,242,243,244,245,246]
COG7	UNQ3082/PRO10013	Intra-Golgi retrograde trafficking	Yes (‘N’ and ‘O’ glycosylation defects)	IVS1 + 4 A → C aka c.169 + 4A > C, c.170-7A > G (p.56–57insAT)	[247,248,249,250]
COG8		Intra-Golgi retrograde trafficking	Yes (‘N’ and ‘O’ glycosylation defects)	c.1611C > G (p.Y537X), IVS3 + 1G > A, TT 1687–1688, c.171dupG (p.L58Afs*29), c.1656dupC (p.A553Rfs*15), c.1583-1G > A	[251,252,253,254]
VPS51	ANG2, C11orf2, C11orf3, FFR	Retrograde transport from endosomes to TGN	Yes (‘N’ and ‘O’ glycosylation defects)	c.1468C > T, c.2232delC (c.1419_1421del; (p (Phe474del)))	[172,255]
VPS52	SACM2L	Retrograde transport from endosomes to TGN	-		
VPS53	Hit1	Retrograde transport from endosomes to TGN	Yes (‘N’ and ‘O’ glycosylation defects)	c.2084A > G c.1556+5G > A	[167]
VPS54	CGP1, LUV1, RKI1, TCS3	Retrograde transport from endosomes to TGN	Yes (‘N’ and ‘O’ glycosylation defects)		
STX5		Intra-Golgi retrograde transport	Yes (‘N’ and ‘O’ glycosylation defects)	c.163A > G (p.(Met55Val))	[197]
GOSR1	GS28	Intra-Golgi retrograde transport	-	-	
GOSR2	GS27/membrin	ER-Golgi transport	-	c.430G > T (p.Gly144Trp)	[196,256,257]
YKT6		Golgi organization and autophagy			
SNAP29		Golgi trafficking and autophagy	-	22q11.2 deletion	[208,210]
STX16		Endosome to TGN transport and maintaining Golgi structure	-	3kb deletion in STX16 gene	[211]

## Data Availability

Not applicable.
